# First diagnosed invasive lobular carcinoma of the breast combined with gastric metastasis and bone metastasis: a case report and review of the literature

**DOI:** 10.1186/s12905-023-02267-6

**Published:** 2023-03-25

**Authors:** Lin Sun, Jiajia Liu, Meng Guo, Jiaqi Xu, Dan Wang

**Affiliations:** Department of Breast Surgery, Second Affiliated Hospital of Jilin University, Changchun, 130041 China

**Keywords:** Breast cancer, Gastrointestinal metastases, Immunohistochemistry, Diagnosis, Treatment, Prognosis

## Abstract

**Rationale:**

Chinese women topped the list of new breast cancers, the first diagnosed gastric metastasis and bone metastasis is extremely infrequent. The clinical and pathological diagnosis of metastatic breast cancer is difficult. To our knowledge, this is the first reported case of the first diagnosis of breast cancer with both gastric metastasis and bone metastasis.

**Case report:**

The female patient was found to have abdominal distension for 15 days with nausea and vomiting. The patient underwent a gastroscopy at an outside hospital 4 days ago, showing: duodenal bulb changes, gastric retention and chronic non-atrophic gastritis. Gastroscopic biopsy showed chronic inflammation and edema of the duodenal mucosa with glandular hyperplasia. Conservative treatment was given with no relief of symptoms. She was seen in our hepatobiliary and pancreatic surgery department. After admission, palliative surgery was performed, and the swelling and surrounding involved tissues were taken for examination during surgery. The rapid pathological return could not exclude tumor lesions, and the postoperative pathology confirmed the diagnosis of invasive lobular carcinoma of the breast with gastric metastases, and the systemic examination revealed combined bone metastases.

**Diagnosis:**

Pathology and immunohistochemistry(IHC), a whole-body bone scan confirmed the first diagnosis of breast cancer with both gastric and bone metastases.

**Interventions:**

Palliative treatment with bisphosphonates and CDK4/6i (Palbociclib) in combination with AI (Exemestane) was administered.

**Outcomes:**

The patient is currently under regular evaluation and is being followed up.

**Supplementary Information:**

The online version contains supplementary material available at 10.1186/s12905-023-02267-6.

## Introduction

In February 2022, the latest data from the National Cancer Center showed that Chinese women topped the list of new breast cancers and also reminded women that they should be screened for breast cancer through regular physical examinations. Despite the expanding scope of screening, 3.6% to 6% of newly diagnosed breast cancers are still first diagnosed stage IV [1], with bone (66.3%), lung (30.3%), liver (26.1%), and brain (7.3%) were the most common distant metastatic sites [2], and gastric metastases were extremely rare, with an incidence of about 0.3% [3]. Metastatic breast cancer can have a single tumor in a metastatic organ as the first symptom, which increases the difficulty of clinical and pathological diagnosis. Therefore, this paper reports a patient with gastric metastasis of breast cancer diagnosed with abdominal distension as the first symptom and reviews the related literature, aiming to investigate the clinicopathological features of gastric metastasis of breast cancer and its diagnosis, treatment, and prognosis.

## Case presentation

**Fig. 1:**
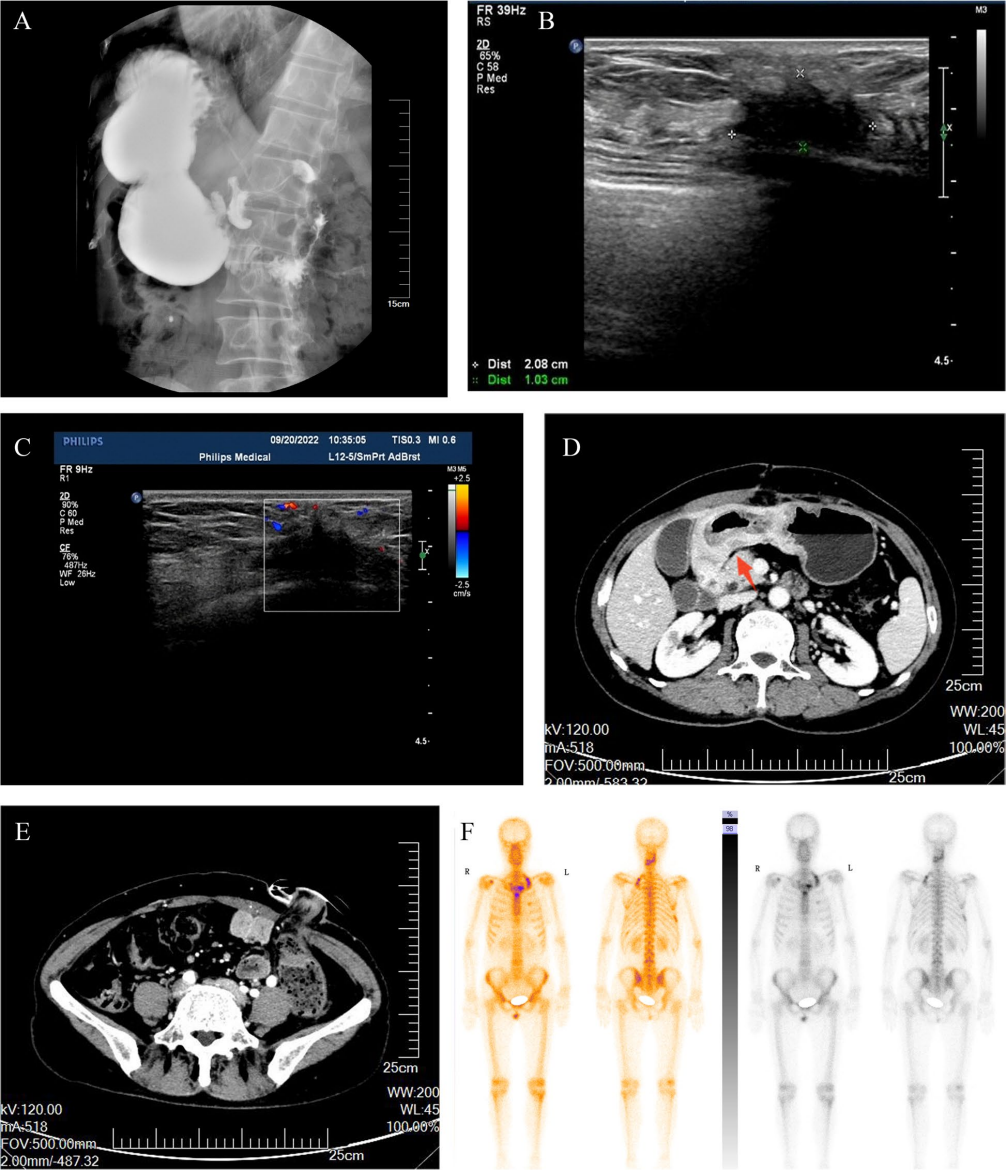
Preoperative upper gastrointestinal X-ray barium meal showed gastric retention, gastroduodenal anastomosis stenosis, and obstructed contrast passage (**A**). Breast ultrasound showed a hypoechoic cluster of about 2.1 cm*1.0 cm in size, with poorly defined and irregular shape, and dotted strong echogenicity in the right breast next to the nipple at point 7, and CDFI: blood flow signal was visible (**B**, **C**). Postoperative gastric CT showed thickening of the gastric wall in the sinus, the thickest part of which was about 22 mm in diameter, involving the whole circumference of the lumen, with a length of about 150 mm, and the surrounding lymph nodes showed that the obstructive symptoms were significantly relieved after surgery (**D**, **E**). The whole-body bone scan suggested multiple tumor metastases (**F**)

**Fig. 2:**
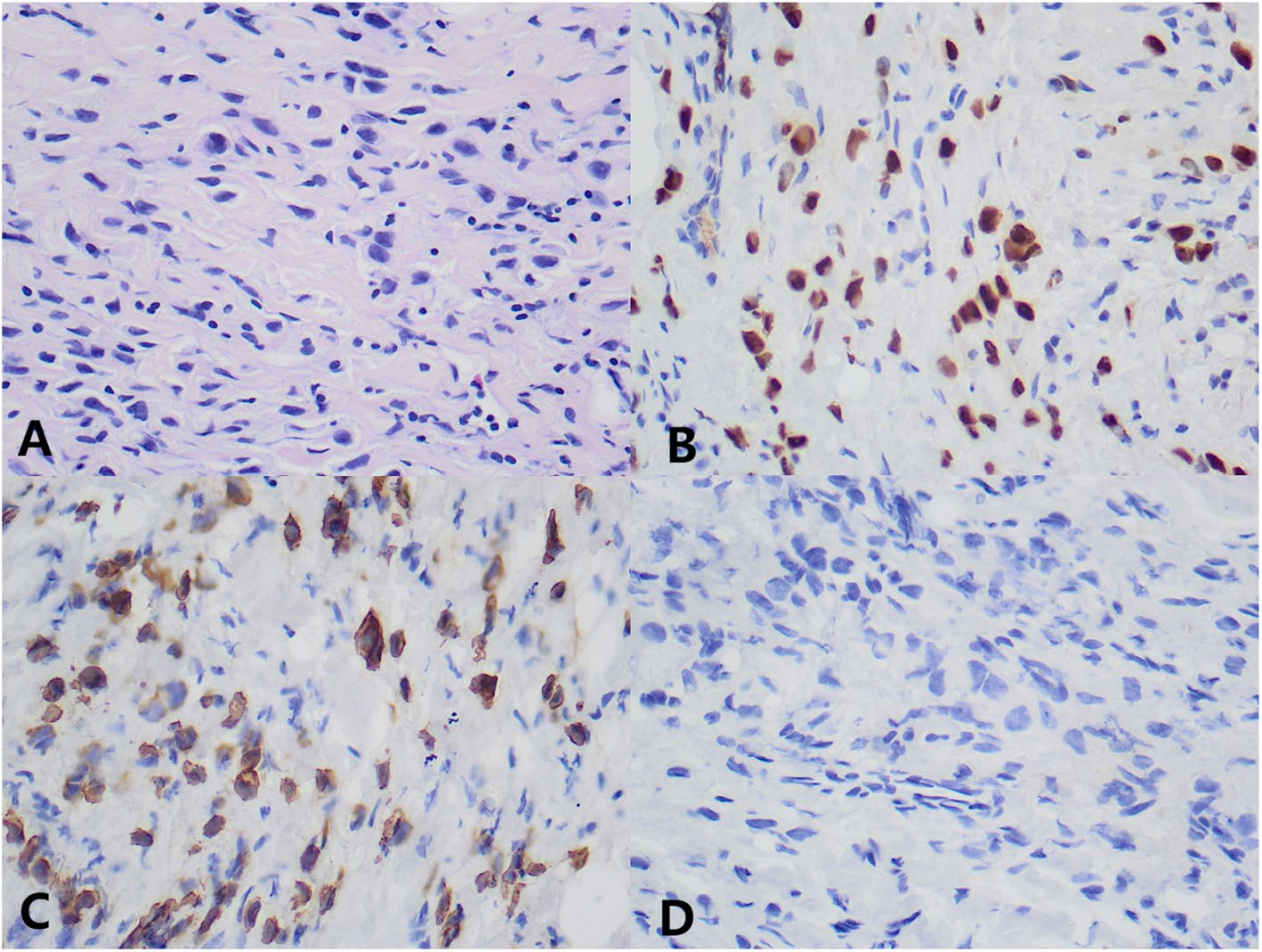
Immunohistochemical picture of mucosal histopathology of gastric metastatic lesions. **A** Original magnification × 400: HE staining shows invasive lobular carcinoma in the metastatic lesion in the gastric sinus. **B** Original magnification × 400: Tumor cells in the gastric metastatic lesion showing positivity for GATA3 by IHC analysis. **C** Original magnification × 400: tumor cells in the gastric metastatic lesion showing positivity for CK7 by IHC analysis. **D** Original magnification × 400: tumor cells in the gastric metastatic lesion showed positive for CK20 by IHC analysis

**Fig. 3:**
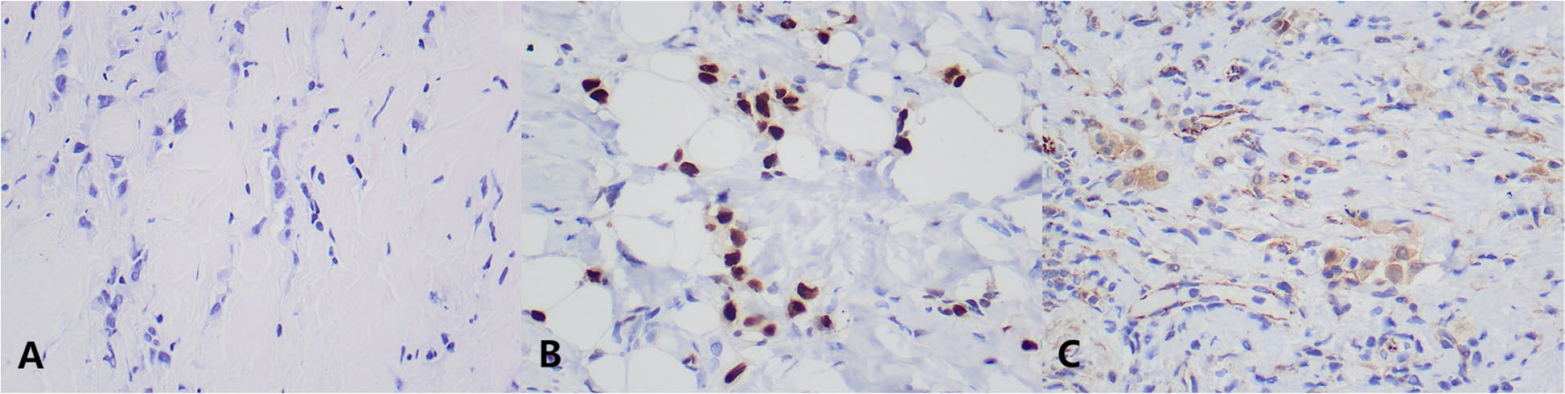
Histopathological immunohistochemical picture of the breast tumor. **A** Original magnification × 400: HE staining shows the pathological type of breast tumor as invasive lobular carcinoma. **B** Original magnification × 400: Breast tumor cells showed positivity for GATA3 by IHC analysis. **C** Original magnification × 400: Breast tumor cells showed positive for intracytoplasmic P120 by IHC analysis

The patient was a 58-year-old female, who was found to have abdominal distension with nausea and vomiting for 15 days. She was seen in our hepatobiliary and pancreatic surgery department on September 5, 2022. The patient had undergone a gastroscopy on September 1, 2022, at an outside hospital, which showed: duodenal bulb changes and gastric retention and chronic non-atrophic gastritis. Gastroscopic biopsy revealed chronic inflammation, edema and glandular hyperplasia in the duodenal mucosa. She received conservative treatment, but her symptoms did not resolve and she was referred to our hospital. The physical examination found no abnormalities or family history of hereditary disease. Barium radiography showed an increase in gastric volume, gastric retention, narrowing of the gastroduodenal anastomosis, and obstructing the passage of the contrast medium (Fig. 1A). The initial diagnosis given to her by our hospital was a duodenal mass with gastric retention. In order to relieve her obstructive symptoms, we performed gastrojejunostomy and colostomy for her on September 15, 2022, and during the operation we saw a localized elevation from the gastric sinus to the duodenal bulb with a size of about 6.0 cm*3.0 cm*2.0 cm during open exploration, the gastric wall where the mass was located was thickened, hard and poorly mobile, and the mass invaded part of the duodenal wall, and continued exploration of the adjacent organs and the lymph nodes were not abnormal, so the involved tissues around the duodenum were taken for examination. Rapid intraoperative pathology showed a small amount of inflammatory cell infiltration in the gastric sinus and a small amount of heterotypic cells, which could not exclude neoplastic lesions, pending further analysis of the slow pathology and immunohistochemistry results. Postoperative pathology reported scattered heterogeneous cells in the fibrous connective tissue of the gastric sinus, combined with immunohistochemical staining results consistent with carcinoma, not excluding invasive lobular carcinoma of mammary gland origin. Immunohistochemical staining results: ER (90%), PR (-), Ki67 (2%), GATA3 ( +), CK (AE1/AE3) ( +), CK7 ( +), CK20 (-) (Fig. 2). The ultrasound examination of both breasts and axillae was completed, and the results showed that a hypoechoic cluster of about 2.1 cm*1.0 cm in size was seen next to the nipple at point 7 of the right breast, with poorly defined and irregular morphology, and dotted strong echogenicity was seen within it. Echogenicity of the right axillary lymph node was observed, with a height of approximately 1.2 cm*0.7 cm, and cortical echogenicity predominated. Ultrasound diagnosis: right breast mass, BIRADS-US classification: class 4C. Right axillary lymph node enlargement (Fig. 1B,C). On September 21, 2022, a coarse needle aspiration biopsy of the right breast mass and axillary lymph nodes was performed under local anesthesia. Puncture pathology results: (right breast mass puncture) invasive lobular carcinoma (classic), (right axillary lymph node puncture) no carcinoma seen. Immunohistochemical results: ER (95%), PR (-), HER-2 (0), Ki67 (5%), GATA3 ( +), E-Cadherin (-), CK (AE1/AE3) ( +), P120 (cytoplasmic +)(Fig. 3). She came to our department for the next treatment on October 8, 2022. After admission, breast tumor markers, whole-body bone scans, and plain gastric CT with enhancement were completed to further evaluate the patient's condition. Breast tumor marker results showed: CA125 value 39.60U/ml (0 ~ 35U/ml), CA153value 32.40U/ml (0 ~ 31.3U/ml); postoperative gastric computed tomography (CT) showed thickening of the gastric wall in the sinus region with surrounding lymph nodes (Fig. 1D,E); whole-body bone scan showed multiple tumor bone metastases, through the whole-body examination and orthopedic specialist consultation, the final consideration was from the breast (Fig. 1F). Diagnosis: right breast cancer, cT2N0M1, stage IV. Treatment: palliative treatment with bisphosphonates and CDK4/6i (Palbociclib) combined with AI (Exemestane). The patient is currently under regular evaluation and is being followed up.

## Discussion

The patient in this report was initially diagnosed with gastric metastasis, and the final diagnosis was revised to gastric metastasis and bone metastasis from breast cancer after the completion of relevant tests. Gastric metastases from breast cancer mostly occur years after the diagnosis of breast cancer, and Ayantunde et al. found that the mean latency period between the diagnosis of primary breast cancer and GI metastases was 6.5 years through a review of databases [4]. Its clinical presentation is mostly dyspepsia and epigastric pain, which may be accompanied by bleeding and nausea, and vomiting, so it is often difficult to distinguish from primary gastric cancer. The majority (94%) of patients will also have metastases from other sites, mainly in the bone [5]. The gastroscopic presentation of gastric metastases from breast cancer is usually a diffuse infiltrative inflammation of the submucosa and intrinsic muscular layer, while scattered nodules and external compression are infrequently seen [5]. Diffuse infiltration is in turn characteristic of invasive lobular carcinoma metastases, and Taal et al. reported that 83% of patients with gastric metastases from breast cancer had lobular breast cancer as the predominant pathology [5]. Therefore, patients with invasive lobular carcinoma of the breast should be alerted to the presence of gastric metastases when they develop gastrointestinal symptoms. Moreover, the reported rate of diagnosis of gastrointestinal or peritoneal metastases in advanced breast cancer is 6% in living patients and 31% in autopsy specimens. The higher rate obtained in autopsy specimens may be due to the fact that candidates for autopsy are patients who have experienced peritoneal dissemination and gastrointestinal metastases, which are usually fatal to patients. In addition, this may be why abdominal examination may not always be emphasized during postoperative follow-up of breast cancer [6, 7]. In this case, the patient had abdominal distension as the first symptom, and the examination results suggested a huge nodule in the gastric sinus, relying on intraoperative and postoperative pathology to make the diagnosis, a situation rarely mentioned in all reports so far.

**Fig. 4:**
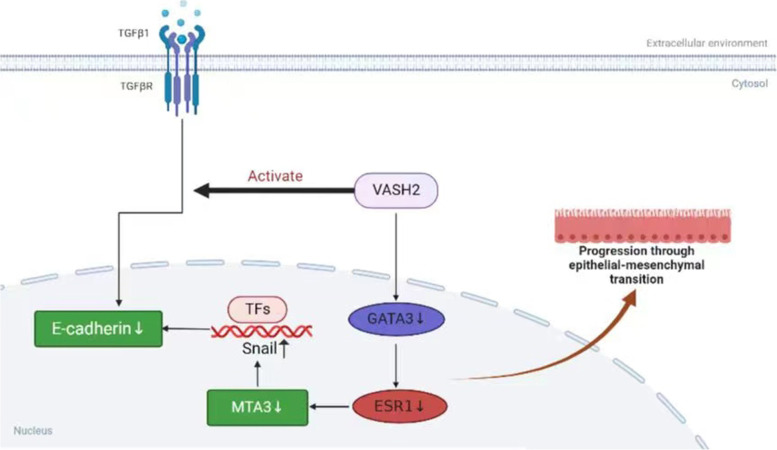
VASH2 induces EMT pathway: VASH2 induces TGFβ1 expression in vitro and in vivo, and the activated TGFβ pathway downregulates E-cadherin. VASH2 also decreases E-cadherin expression by downregulating GATA3 expression under hypoxic conditions in vitro or in vitro, GATA3 is an ESR1 transcription factor, and the reduction of GATA3 transcript level GATA3 is an ESR1 transcription factor, and the reduction of GATA3 transcript level resulted in the down-regulation of ESR1

Pathological biopsy remains the gold standard for the diagnosis of gastric metastasis from breast cancer. It is usually difficult to identify the origin of the tumor by gastroscopy and imaging performance alone, and gastroscopic biopsy often results in false negatives, as in the present case, where the initial gastroscopic biopsy produced false negative results. The combination of clinical history, clinical symptoms, and pathological changes sometimes fail to identify the tumor at the initial diagnosis, which reflects the importance of immunohistochemical staining. It has been reported that even if the primary site of breast cancer are positive for estrogen receptor (ER) and progesterone receptor (PR) expression, gastric metastases can still be negative for estrogen receptor (ER) and progesterone receptor (PR) status [8]; therefore, the diagnosis of breast cancer metastases by hormone receptor expression status is not yet reliable. Studies have shown that the lack of E-calciferol receptor (E-cadherin) expression was significantly correlated with metastatic breast invasive lobular carcinoma, and the absence of E-cadherin expression in gastroscopic cancer tissues may increase the likelihood of breast invasive lobular carcinoma metastasis [9, 10]. So how is E-cadherin involved in the process of breast cancer metastasis? It was found that the epithelial-mesenchymal transition pathway (EMT) plays an important role in the metastasis of malignant tumors such as breast, cervical, lung, and gastric cancers [11–13], and vasopressor 2 (VASH2), a proto-oncogene, was shown to inhibit E-cadherin by activating transforming growth factor β1 (TGFβ1) and hypoxia-dependent GATA3 binding protein (GATA3) thereby inhibiting cadherin expression to promote EMT in human breast cancer (Fig. 4). In addition, GATA-binding protein 3 (GATA3) and vesicular disease fluid protein-15 (GCDFP-15) are useful in the diagnosis of breast cancer metastases and can also play an important role in the differential diagnosis, and positive expression of GCDFP-15 is a highly sensitive (55–76%) and specific (95–100%) marker that can correctly identify malignant lesions identified as metastatic breast cancer [14–16]. Studies have shown that GATA3 has a diagnostic sensitivity of 32–90%, is localized in the nucleus, and is positive in all breast tumor cells, whereas GCDFP-15 is positive in only a subset of tumor cells, and GATA3 expression is extremely low in gastrointestinal cancers, so the identification of breast cancer gastric metastases with GATA3 antibody would be very effective [17]. The detection of cytokeratin 7 (CK7) and cytokeratin 20 (CK20) also contributes to tumor identification. Breast cancer is usually positive for CK7 expression and negative for CK20 expression, in addition, positive CK7 expression and negative CK20 expression are also present in ovarian, lung, and thyroid cancers, and CK20 is usually expressed in colorectal and other gastrointestinal cancers, and rarely positive in breast cancer [18–20]. In this case, the immunohistochemistry of gastric metastasis was ER (90%), PR (-), Ki67 (2%), GATA3 ( +), CK (AE1/AE3) ( +), CK7 ( +), and CK20 (-), suggesting that the tumor originated from the breast and was considered as gastric metastasis of invasive lobular carcinoma of the breast. In conclusion, the diagnosis of a patient with metastatic breast cancer should combine clinical presentation, imaging, endoscopy, pathological changes, and immunohistochemical analysis, and the immunohistochemical analysis should use a set of immunohistochemical antibodies because no marker is 100% specific or sensitive. Also, a detailed history of the patient's breast cancer makes it more likely that the diagnosis will be missed because of failure to perform breast cancer-related antibodies.

There is no guideline for the treatment of breast cancer metastases in the stomach. At present, systemic treatment such as chemotherapy and endocrine therapy is the main objective to prolong patients' survival and improve their quality of life. Among them, CDK4/6 inhibitors belong to a group of targeted therapies, mainly targeting combined hormone therapy for hormone receptor-positive HER2-negative advanced breast cancer. In patients with advanced hormone receptor-positive breast cancer, the PALOMA-2 clinical trial showed that palbociclib combined with an aromatase inhibitor (AI) as first-line endocrine therapy prolonged progression-free survival (PFS) by 10.3 months compared to the control group [21]. Surgery is not the first choice and is mostly used for palliative treatment when patients have complications such as obstruction, bleeding, and perforation. The prognosis of gastric metastases from breast cancer is poor, and some data show that the average survival of all women with metastatic disease secondary to breast cancer is 24 to 36 months [22], but the author found that the majority (84%) of patients included in the data of these studies had GI metastases years after the diagnosis of primary breast cancer had been made. This patient was diagnosed with both primary breast cancer, gastric metastases, and bone metastases at the initial diagnosis, which also suggests a worse prognosis. Because the patient had advanced breast cancer and had undergone gastrointestinal palliative surgery, endocrine therapy with CDK4/6 inhibitors in combination with exemestane was selected after a multidisciplinary consultation.

## Conclusion

Gastric metastases from breast cancer are unusual, and it is even rarer to diagnose primary breast cancer combined with gastric and bone metastases at the time of initial diagnosis. To the best of our knowledge, this is the first report of a first diagnosis of breast cancer with both gastric and bone metastases, and close follow-up will be performed to obtain survival data for this patient, aiming to provide more data support for future studies. We hope that more such cases will be reported in the future to enrich the clinicians' experience and provide better survival benefit to this group of patients.

## Supplementary Information

**Additional file 1.**

## Data Availability

If anyone would like to obtain data from this study, please contact the corresponding author: Dan Wang,wangd99@jlu.edu.cn.

## Abbreviations

GATA3: GATA-binding protein 3
IHC: Immunohistochemistry
CK7: Cytokeratin 7
CK20: Cytokeratin 20
ER: Estrogen receptor
PR: Progesterone receptors
Her-2: Human epidermal growth factor 2
AI: Aromatase inhibitor
CDK4/6i: Cyclin-dependent kinase 4/6 inhibitor
VASH2: Vasohibin 2
TGFβ1: Transforming growth factor β1
ESR1: Estrogen receptor 1

## References

[1] Siegel RL, Miller KD, Jemal A (2017). Cancer Statistics, 2017. CA Cancer J Clin.

[2] Wang H, Zhang C, Zhang J (2017). The prognosis analysis of different metastasis patterns in patients with different breast cancer subtype: a SEER based study. Oncotarget.

[3] Rodrigues MV, Tercioti-Junior V, Lopes LR (2016). Breast Cancer Metastasis in the Stomach: When the Gastrectomy Is Indicated ?. Arq Bras Cir Dig.

[4] Ayantunde AA, Agrawal A, Parsons SL (2007). Esophagogastric cancers secondary to a breast primary tumor do not require resection. World J Surg.

[5] Taal BG, Peterse H, Boot H (2000). Clinical presentation, endoscopic features, and treatment of gastric metastases from breast carcinoma. Cancer.

[6] Tsubono M, Kaneko I, Kii E, Tanaka T, Murata T, Kamimura K (2005). Weekly Paclitaxel administration and intraabdominal CBDCA injection possibly beneficial treatment for recurrent breast cancer associated with metastatic ovarian cancer and peritoneal dissemination after operation—a case report. Jpn J Cancer Chemother.

[7] Sumi T, Mochizuki M, Katsumata K, Ri M, Suzuki Y, Kato F (2005). A case of peritoneal metastasis of breast cancer after operation. JJCS.

[8] Kim DH, Son SM, Choi YJ (2018). Gastric metastasis from invasive lobular breast cancer, mimicking primary gastric cancer: a case report. Medicine (Baltimore).

[9] van Velthuysen ML, Taal BG, van der Hoeven JJ (2005). Expression of oestrogen receptor and loss of E-cadherin are diagnostic for gastric metastasis of breast carcinoma. Histopathology.

[10] Sastre-Garau X, Jouve M, Asselain B (1996). Infiltrating lobular carcinoma of the breast: Clinicopathologic analysis of 975 cases with reference to data on conservative therapy and metastatic patterns. Cancer.

[11] Sun X, Lin F, Sun W (2021). Exosome-transmitted miRNA-335-5p promotes colorectal cancer invasion and metastasis by facilitating EMT via targeting RASA1. Mol Ther Nucleic Acids.

[12] Beuran M, Negoi I, Paun S (2015). The epithelial to mesenchymal transition in pancreatic cancer: a systematic review. Pancreatology.

[13] Wang S, Tong X, Li C (2021). Quaking 5 suppresses TGF-beta-induced EMT and cell invasion in lung adenocarcinoma. EMBO Rep.

[14] Bhargava R, Beriwal S, Dabbs DJ (2007). Mammaglobin vs GCDFP-15: an immunohistologic validation survey for sensitivity and specificity. Am J Clin Pathol.

[15] O’Connell FP, Wang HH, Odze RD (2005). Utility of Immunohistochemistry in Distinguishing Primary Adenocarcinomas From Metastatic Breast Carcinomas in the Gastrointestinal Tract. Arch Pathol Lab Med.

[16] Honma N, Takubo K, Arai T (2006). Comparative study of monoclonal antibody B72.3 and gross cystic disease fluid protein-15 as markers of apocrine carcinoma of the breast. APMIS.

[17] Gown AM, Fulton RS, Kandalaft PL (2016). Markers of metastatic carcinoma of breast origin. Histopathology.

[18] Lee AH (2007). The histological diagnosis of metastases to the breast from extramammary malignancies. J Clin Pathol.

[19] Chu PG, Weiss LM (2002). Keratin expression in human tissues and neoplasms. Histopathology.

[20] Lee AHS, Hodi Z, Soomro I (2020). Histological clues to the diagnosis of metastasis to the breast from extramammary malignancies. Histopathology.

[21] Finn RS, Martin M, Rugo HS (2016). Palbociclib and Letrozole in Advanced Breast Cancer. N Engl J Med.

[22] McLemore EC, Pockaj BA, Reynolds C (2005). Breast cancer: presentation and intervention in women with gastrointestinal metastasis and carcinomatosis. Ann Surg Oncol.

