# Supplementary material for: First diagnosed invasive lobular carcinoma of the breast combined with gastric metastasis and bone metastasis: a case report and review of the literature
Source: BMC Womens Health. 2023 Mar 25;23:133. doi: 10.1186/s12905-023-02267-6 (PMC10040113; doi:10.1186/s12905-023-02267-6)
Supplement: Supplementary file 1 — Additional file 1. [file 12905_2023_2267_MOESM1_ESM.docx]

Supplementary File:Highlights of the Manuscript

1. The first diagnosed gastric metastasis is extremely rare.
2. The clinical and pathological diagnosis of metastatic breast cancer is difficult.
3. To our knowledge,this is the first reported case of the first diagnosis of breast cancer with both gastric metastasis and bone metastasis.
